# Molecular interactions from the density functional theory for chemical reactivity: Interaction chemical potential, hardness, and reactivity principles

**DOI:** 10.3389/fchem.2022.929464

**Published:** 2022-07-22

**Authors:** Ramón Alain Miranda-Quintana, Farnaz Heidar-Zadeh, Stijn Fias, Allison E. A. Chapman, Shubin Liu, Christophe Morell, Tatiana Gómez, Carlos Cárdenas, Paul W. Ayers

**Affiliations:** ^1^ Department of Chemistry and Quantum Theory Project, University of Florida, Gainesville, FL, United States; ^2^ Department of Chemistry, Queen’s University, Kingston, ON, Canada; ^3^ Department of Chemistry and Chemical Biology, McMaster University, Hamilton, ON, Canada; ^4^ Research Computing Center, University of North Carolina, Chapel Hill, NC, United states; ^5^ Université de Lyon, Universit́e Claude Bernard Lyon 1, Institut des Sciences Analytiques, UMR CNRS 5280, Villeurbanne Cedex, France; ^6^ Theoretical and Computational Chemistry Center, Institute of Applied Chemical Sciences, Faculty of Engineering, Universidad Autonoma de Chile, Santiago, Chile; ^7^ Departamento de Fisica, Facultad de Ciencias, Universidad de Chile, Santiago, Chile; ^8^ Centro para el desarrollo de la Nanociencias y Nanotecnologia, CEDENNA, Santiago, Chile

**Keywords:** DFT‐density functional theory, chemical reactivity, HSAB (hard-soft-acid-base) concept, chemical potential, variational principle

## Abstract

In the first paper of this series, the authors derived an expression for the interaction energy between two reagents in terms of the chemical reactivity indicators that can be derived from density functional perturbation theory. While negative interaction energies can explain reactivity, reactivity is often more simply explained using the “|dμ| big is good” rule or the maximum hardness principle. Expressions for the change in chemical potential (*μ*) and hardness when two reagents interact are derived. A partial justification for the maximum hardness principle is that the terms that appear in the interaction energy expression often reappear in the expression for the interaction hardness, but with opposite sign.

## 1 Introduction

Nearly 35 years ago, Parr recognized that density-functional theory (DFT) could be used not only as a formal alternative to wavefunction-based quantum chemistry and as a computational tool, but also as an interpretative tool through which chemical reactivity could be elucidated (Parr et al., 1978; Parr and Yang, 1989). The special power of the electron-density perspective arises because the mathematical structure of DFT naturally accommodates fractional numbers of electrons and therefore partial electron transfer (Janak, 1978; Parr and Bartolotti, 1982; Perdew et al., 1982; Yang et al., 2000; Ayers, 2008; Fuentealba and Cardenas, 2013; Miranda-Quintana and Bochicchio, 2014; Miranda-Quintana and Ayers, 2016a; Ayers and Mel, 2018), while in the wavefunction theory, the number of electrons is linked to the dimensionality of the wavefunction, and is inherently an integer. Ironically, the great utility of DFT for conceptual purposes arises from the same feature that is most problematic for computational applications (Merkle et al., 1992; Mori-Sanchez et al., 2006; Ruzsinszky et al., 2007; Cohen et al., 2008a; b;Mori-Sanchez et al., 2008; Yang et al., 2016). However, the difficulty of treating fractional electrons *computationally* in DFT is not important in the context of this paper, where one can assume that an exact (or otherwise accurate *ab initio*) density functional is used (Levy, 1979; Lieb, 1983; Bartlett et al., 2005; Ayers, 2006).

The use of density functional theory for chemical reactivity (DFT-CR), often called conceptual DFT or chemical DFT (Parr and Yang, 1989; Chermette, 1999; Geerlings et al., 2003; Ayers et al., 2005; Chattaraj et al., 2006; Gazsquez, 2008; Liu, 2009a; Johnson et al., 2012; De Proft et al., 2014; Fuentealba and Cardenas, 2015; Miranda-Quintana, 2018), is now well established, both mathematically, conceptually, and computationally. The greatest successes of DFT-CR are probably linked to the definition of a quantitative scale for the chemical hardness, (Parr and Pearson, 1983), which has led to increased understanding of the hard/soft acid/base (HSAB) theory (Nalewajski, 1984; Nalewajski et al., 1988; Chattaraj et al., 1991; Gazquez and Mendez, 1994; Mendez and Gazquez, 1994; Chattaraj, 2001; Melin et al., 2004; Ayers, 2005; Ayers et al., 2006; Anderson et al., 2007a; Ayers, 2007; Cardenas and Ayers, 2013; Miranda-Quintana R. A., 2017) and an entirely new principle for chemical reactivity and molecular stability, the maximum hardness principle (MHP) (Pearson, 1987; Zhou and Parr, 1989; Parr and Chattaraj, 1991; Pearson and Palke, 1992; Pearson, 1994; Chattaraj, 1996; Pearson, 1999; Ayers and Parr, 2000; Torrent-Sucarrat et al., 2001, 2002; Miranda-Quintana R. A., 2017). One of the subtlest results in DFT-CR is that the HSAB principle is, in fact, an inexorable consequence of the maximum hardness principle (Chattaraj et al., 1991; Parr and Chattaraj, 1991; Chattaraj and Ayers, 2005; Chattaraj et al., 2007), with a similar result also connecting the HSAB and minimum electrophilicity principles (Chattaraj and Nath, 1994; Chattaraj et al., 2001; Chattaraj, 2007; Noorizadeh, 2007; Liu, 2009b; Morell et al., 2009; Pan et al., 2013; Miranda-Quintana, 2017a; Miranda-Quintana and Ayers, 2018b; 2019). Here it is important to clarify that what is usually understood as a “principle” in chemistry is different from the definition in physics. For example, Heisenberg’s uncertainty principle does not admit violations. The principles of chemical reactivity, on the other hand, may have exceptions. Therefore, instead of speaking of principles, one should speak of “rules of thumb” for chemical reactivity. However, for consistency with the literature, here we will use both terms as synonyms.

Almost all applications and theory in DFT-CR have been based on a one-reagent approach: the response functions of a reactant molecule are computed, and then used to predict its reactivity. Despite the usefulness of this approach, it sometimes fails. That is, sometimes understanding the inherent reactivity of a molecule is insufficient; one must discern how well-matched two reagents are. Early attempts at quantifying well-matched-ness were made by Berkowitz, Geerlings, and then, much later, by one of the present authors (Berkowitz, 1987; Langenaeker et al., 1995; Ayers et al., 2006; Anderson et al., 2007a; Ayers, 2007; Anderson et al., 2007b; Ayers and Cardenas, 2013). In the first paper of this series (Miranda-Quintana et al., 2022b), we derived a general expression for the interaction energy between two reagents using DFT-CR and drew the links to other, more computational, DFT theories like density-functional embedding (Cortona, 1991; Vaidehi et al., 1992; Wesolowski and Warshel, 1993; Govind et al., 1999; Wesolowski, 2004; Wesolowski and Leszczynski, 2006), electronegativity equalization molecular mechanics (Yang and Mortier, 1986; Mortier et al., 1985; Mortier et al., 1986; Yang and Mortier, s1986; Mortier, 1987; Rappe and Goddard, 1991; Bultinck et al., 2002a; Bultinck et al., 2002b; Verstraelen et al., 2013), and density-based energy decomposition analysis (Wu et al., 2009).

While the interaction energy provides the most fundamental perspective on chemical reactivity, sometimes it is simpler to understand chemical reactivity using alternative reactivity rules. For example, the “|dμ| big is good” (DMB) rule of Parr and Yang (Parr, 1994; Miranda-Quintana R. A. and Ayers P. W., 2018; Miranda-Quintana et al., 2018) states that favorable chemical interactions are usually associated with a large change in the chemical potential (Miranda-Quintana et al., 2018; Miranda-Quintana and Ayers, 2019; Miranda-Quintana et al., 2021). Likewise, Sanderson’s electronegativity equalization principle (Mortier et al., 1986) and Pearson’s HSAB (Pearson, 1968a; Pearson, 1968b; Nalewajski, 1984; Nalewajski et al., 1988; Chattaraj et al., 1991; Miranda-Quintana R. A., 2017; Miranda-Quintana R. A. et al., 2017), and the more recent minimum electrophilicity principles (Fuentealba et al., 2000b; Morell et al., 2009; Miranda-Quintana R. A., 2017) put these reactivity descriptors in center stage by telling us how to use them to understand and predict chemical reactivity (Sanderson, 1951; Parr et al., 1978). It is not farfetched to say that the biggest triumph of DFT-CR is not only to provide mathematically precise definitions for the reactivity descriptors, but to also give us a robust framework to derive new ones (Ayers et al., 2018; Geerlings et al., 2020). However, more often than not, these derivations have been solely based on the venerable parabolic model of Parr and Pearson (Parr and Bartolotti, 1982; Parr and Pearson, 1983; Chattaraj et al., 1995; Ayers and Parr, 2008; Alain Miranda-Quintana and Ayers, 2016; Heidar-Zadeh et al., 2016b; Miranda-Quintana and Ayers, 2016b; Cárdenas et al., 2016; Franco-Pérez et al., 2018). While powerful and hugely influential, this model can be seen as the simplest representation of electron transfer during a chemical reaction. This simplicity has often been (rightfully) argued as one of its key advantages, but this also means that elementary proofs of the HSAB (Pearson, 1968a; Chattaraj et al., 1991), minimum electrophilicity, maximum hardness, and DMB principles usually ignore electrostatic and polarization effects, and do not include charge transfer effects beyond second order (Chattaraj et al., 1991; Parr and Chattaraj, 1991; Ayers and Parr, 2000; Ayers, 2005; Chattaraj and Ayers, 2005; Ayers and Cardenas, 2013; Miranda-Quintana et al., 2018; Ayers et al., 2022). Recently, some of these approximations have been relaxed (Alain Miranda-Quintana et al., 2016; Miranda-Quintana and Ayers, 2016a; Miranda-Quintana R. A., 2017; Miranda-Quintana et al., 2021; Miranda-Quintana et al., 2022a), which strengthens the support for these principles, but the more realistic two-reagent picture remains largely unexplored (Ayers et al., 2006; Ayers, 2007; Chattaraj et al., 2007; Miranda-Quintana R. A. et al., 2017).

The chemical potential (Parr et al., 1978),

$$\mu ={\left(\frac{\partial E}{\partial N}\right)}_{v\left(r\right)}$$ (1)

measures the intrinsic Lewis acid/base strength of a molecule and can be considered to be minus one times the electronegativity. The essence of the rule, then, is that favorable molecular interactions between acids and bases “quench” the acidity/basicity of the reagents as much as possible (forming, in the extreme case, nearly inert salts). Alternatively, favorable chemical changes are associated with large changes in molecular electronegativity. Even though the “|dμ| big is good” rule was first formulated more than 30 years ago, its theoretical provenance has only recently begun to be elucidated (Miranda-Quintana et al., 2018; Miranda-Quintana and Ayers, 2019; Miranda-Quintana et al., 2021).

The maximum hardness principle (MHP) is a particularly interesting case, with roots that seem less transparent than many of the aforementioned reactivity rules. (Pearson, 1987; Pearson and Palke, 1992; Pearson, 1993; Chattaraj, 1996; Pearson, 1999). The maximum hardness principle indicates that more stable conformations are associated with a large hardness,

$$\eta ={\left(\frac{\partial^{2}E}{\partial N^{2}}\right)}_{v\left(r\right)}$$ (2)

A corollary of this principle is that the harder a molecule is, the more stable it is. The problem is that the mathematical assumptions under which the maximum hardness principle has been proved (fixed molecular geometry and either constant electron number or constant chemical potential) do not match the conditions under which the principle is usually applied, because the MHP is most commonly used to study molecular rearrangements (Torrent-Sucarrat et al., 2001; 2002). For example, there has been substantial recent interest in using the initial hardness response (the change in hardness associated with the initial approach of two reagents) to study pericyclic reactions (De Proft et al., 2006; Ayers et al., 2007; De Proft et al., 2008; Geerlings et al., 2012).

In the remainder of this paper, we differentiate the energy expression in Eq. 33 of the first paper in this series with respect to the number of electrons. This gives the change in chemical potential (related to the first derivative) hardness (second derivative) due to the interactions between two reagents. These expressions are then used to mathematically justify the “|dμ| big is good” and maximum hardness principles.

The key expression [Eq. 33 from (Miranda-Quintana et al., 2022b)] is

In this equation, Δ*U*_AB_ is the change in the total energy (*U* = E + *V*_*nn*_, where *V*_*nn*_ is the nuclear-nuclear repulsion energy) when the reagents *A* and *B* come together. Δ*N* is the change in the number of electrons in the reagent. Superscript “0” indicates that the term is evaluated for the isolated reagent; subscripts index the reagents. As explained in (Miranda-Quintana et al., 2022b), Eq. 3 can be iterated. The only difference between Eq. 3 and the equation Eq. 33 in (Miranda-Quintana et al., 2022b), is that last it was assumed that $\Delta N_{A}=-\Delta N_{B}$ . That is, every electron that leaves one reagent goes to the other reagent, and the number of electrons in the combined system does not change. In this paper, we will extend this analysis to consider changes in the total number of electrons,

$$\Delta N=\Delta N_{A}+\Delta N_{B}$$ (4)

Most of the reactivity indicators that enter into Eq. 3 are well-known in DFT-CR: the Fukui function *f*(**r**) (Parr and Yang, 1984; Yang et al., 1984; Ayers P. W. and Levy M., 2000; Heidar-Zadeh et al., 2016a; Fuentealba et al., 2016), the dual descriptor *f* ^(2)^(**r**) (Fuentealba and Parr, 1991; Morell et al., 2005, 2006; Ayers et al., 2007; Cardenas et al., 2009b; Geerlings et al., 2012), and the electron density ρ(**r**). The change in energy and density upon polarization of one reagent by another are defined through,

$$\begin{array}{c} \Delta E_{\text{pol}}\approx \frac{1}{2}\iint \Delta v\left(r\right)\Delta v\left(r^{\prime }\right)\chi \left(r,r^{\prime }\right)drdr^{\prime }\\ \approx \frac{1}{2}\int \Delta \rho^{\left(\text{pol}\right)}\left[\Delta v;r\right]\Delta v\left(r\right)dr. \end{array}$$ (5)

where χ(**r**,**r’**) is the linear-response (or polarizability) kernel χ(**r**,**r’).** For convenience, Eq. 3 is written in terms of the nuclear charge density instead of the external potential, (Ayers et al., 2009)

$$v\left(r\right)=-\int \frac{z\left(r^{\prime }\right)}{\left|r-r^{\prime }\right|}dr^{\prime }$$ (6)

The molecular electrostatic potential (Politzer, 1980; Politzer and Truhlar, 1981; Sjoberg and Politzer, 1990; Gadre et al., 1992; Shirsat et al., 1992; Murray et al., 1996; Suresh and Gadre, 1998; Politzer and Murray, 2002),

$$\Phi \left(r\right)=\int \frac{z\left(r^{\prime }\right)-\rho \left(r^{\prime }\right)}{\left|r-r^{\prime }\right|}dr^{\prime }$$ (7)

is not traditionally considered a reactivity indicator in DFT-CR, but it can be placed in a DFT context by differentiating the total energy (including *V*_*nn*_) with respect to the external potential (Ayers and Parr, 2001; Anderson et al., 2007a). The non-additive kinetic and exchange-correlation energies in the first line of Eq. 3 capture electron-pairing and steric effects, (Gordon and Kim, 1972; Wesolowski and Warshel, 1993; Wesolowski and Warshel, 1994; Liu, 2007; Wu et al., 2009)

$$T_{s}^{\text{non}-\text{add}}\left[\rho_{A},\rho_{B}\right]\equiv T_{s}\left[\rho_{AB}\right]-T_{s}\left[\rho_{A}\right]-T_{s}\left[\rho_{B}\right]$$ (8)

$$E_{xc}^{\text{non}-\text{add}}\left[\rho_{A},\rho_{B}\right]\equiv E_{xc}\left[\rho_{AB}\right]-E_{xc}\left[\rho_{A}\right]-E_{xc}\left[\rho_{B}\right].$$ (9)

### 1.1 The interaction hardness and chemical potential

To elucidate the MHP, we need to compute the change in hardness due to the interactions between *A* and *B*. To do this, we define the interaction hardness

$$\Delta \eta_{AB}=\eta_{AB}-\eta_{A}^{0}-\eta_{B}^{0}$$ (10)

and notice that this quantity can be computed by differentiating the interaction energy,

$$\Delta \eta_{AB}={\left(\frac{\partial^{2}\Delta U_{AB}}{\partial N^{2}}\right)}_{v_{AB}\left(r\right)}={\left(\frac{\partial^{2}U_{AB}}{\partial N^{2}}\right)}_{v_{AB}\left(r\right)}-{\left(\frac{\partial^{2}U_{A}^{0}}{\partial N^{2}}\right)}_{v_{A}^{0}\left(r\right)}-{\left(\frac{\partial^{2}U_{B}^{0}}{\partial N^{2}}\right)}_{v_{B}^{0}\left(r\right)}$$ (11)

The treatment of the chemical potential is a bit more nuanced, because since it is an intensive property the correct expression is (Miranda-Quintana et al., 2018):

$$\Delta \mu_{AB}=\mu_{AB}-\frac{1}{2}\left(\mu_{A}+\mu_{B}\right)$$ (12)

with

$$\Delta \mu_{AB}={\left(\frac{\partial \Delta U_{AB}}{\partial N}\right)}_{v_{AB}\left(r\right)}={\left(\frac{\partial U_{AB}}{\partial N}\right)}_{v_{AB}\left(r\right)}-\frac{1}{2}\left\{{\left(\frac{\partial U_{A}^{0}}{\partial N}\right)}_{v_{A}^{0}\left(r\right)}+{\left(\frac{\partial U_{B}^{0}}{\partial N}\right)}_{v_{B}^{0}\left(r\right)}\right\}$$ (13)

The total energy can be used in the previous equations because the nuclear-nuclear repulsion energy does not depend on the number of electrons.

As complicated as it is, Eq. 3 already contains assumptions, most notably assumptions about the “effective external potential” that electrons in one reagent feel due to the electrons and nuclei in the second reagent (Ayers and Parr, 2001; Ayers et al., 2005; Cohen and Wasserman, 2007; Cohen et al., 2009; Liu et al., 2009; Elliott et al., 2010; Osorio et al., 2011). We also must assume that the higher-order terms in the Taylor series (which are implicitly neglected or averaged over in a Taylor-series-with-remainder strategy) are negligible. Extension to include higher-order terms can be made, with commensurate increased complexity in Eq. 3 (Senet, 1996; Geerlings and De Proft, 2008; Cardenas et al., 2009a; Heidar-Zadeh et al., 2016b). Finally, we must assume that the derivatives exist, which implicitly requires that the system is not quasi-degenerate for perturbations of the strength relevant for the analysis. Quasi-degeneracy (even exact degeneracy) can be treated, however, if the derivatives are reinterpreted as differentials (Cardenas et al., 2011; Bultinck et al., 2013a; Bultinck et al., 2013b; Pino-Rios et al., 2017; Cerón et al., 2020; Bultinck and Cárdenas, 2022; Cárdenas et al., 2022). Other effects (e.g., temperature-dependence, spin-specificity) can likewise be treated without essential difficulty, merely by an extension of definition and notation (Galvan et al., 1988; Ghanty and Ghosh, 1994; Ayers and Yang, 2006; Garza et al., 2006; Perez et al., 2008; Franco-Perez et al., 2015a; Franco-Perez et al., 2015b; Alain Miranda-Quintana and Ayers, 2016; Miranda-Quintana R. A. and Ayers P. W., 2016; Franco-Perez et al., 2017a; Franco-Perez et al., 2017b; Franco-Pérez et al., 2017; Robles et al., 2018; Gázquez et al., 2019). The following analysis can also be treated at an atom (or functional-group) condensed level: the integrations over space are merely replaced by sums over atom labels (Yang and Mortier, 1986; Fuentealba et al., 2000a; Ayers et al., 2002; Tiznado et al., 2005; Bultinck et al., 2007; Fuentealba et al., 2016; Echegaray et al., 2017). That provides a more computationally practical form for these results and draws the link to electronegativity equalization methods more strongly.

To obtain expressions for *µ*_*AB*_ and *η*_*AB*_ that are simple enough to be useful, some further assumptions are needed. Before proceeding, let us rewrite Eq. 3 using a shorter, more convenient notation that will help us with the upcoming manipulations:

$$\begin{array}{c} \Delta U_{AB}\left[\Delta N_{A},\Delta N_{B}\right]=\mu_{A}\Delta N_{A}+\mu_{B}\Delta N_{B}+\frac{1}{2}\eta_{A}{\left(\Delta N_{A}\right)}^{2}+\frac{1}{2}\eta_{B}{\left(\Delta N_{B}\right)}^{2}+\\ h_{AB}\Delta N_{A}\Delta N_{B}+\theta_{A}{\left(\Delta N_{A}\right)}^{2}\Delta N_{B}+\theta_{B}\Delta N_{A}{\left(\Delta N_{B}\right)}^{2}+\frac{1}{4}c_{AB}{\left(\Delta N_{A}\Delta N_{B}\right)}^{2} \end{array}$$ (14)

Here *h*_*AB*_ is the Coulomb interaction between the fragments’ Fukui functions, *c*_*AB*_ is the Coulomb interaction between the fragments’ dual descriptors, and θ_*A*_ is the Coulomb interaction between the dual descriptor of fragment *A* and the Fukui function of fragment *B*. To obtain this expression, we just grouped terms according to the powers of $\Delta N_{A},\Delta N_{B}$ , and neglected the terms that are *N*-independent (since we are dealing with derivatives with respect to *N* these terms won’t be relevant). For instance, $\mu_{A}$ , denotes the chemical potential of fragment *A* in the presence of *B* and includes not only a contribution from the chemical potential of the isolated fragment *A*, but also contributions from the interaction of *A* and *B* (e.g., the term $\int f_{A}\left(r\right)\Phi_{B}^{0}\left(r\right)dr$ ). From Eq. 14 it is easy to see that the energy will be minimized if the coefficient of the $\Delta N_{A}\Delta N_{B}$ term is as big as possible (since $\Delta N_{A}\Delta N_{B}<0$ ); while the coefficient of the ${\left(\Delta N_{A}\Delta N_{B}\right)}^{2}$ term is as small as possible (ideally, a negative number). That is:

$$h_{AB}=\iint \frac{f_{A}\left(r\right)f_{B}\left(r^{\prime }\right)}{\left|r-r^{\prime }\right|}drdr^{\prime }>0$$ (15)

$$c_{AB}=\iint \frac{f_{A}^{\left(2\right)}\left(r\right)f_{B}^{\left(2\right)}\left(r^{\prime }\right)}{\left|r-r^{\prime }\right|}drdr^{\prime }<0$$ (16)

In order to evaluate Eq. 11 we need to know the interaction energy as a function of the number of electrons. The number of electrons enters the expression for the interaction energy (Eq. 3) through the reactivity indicators and through the extent of electron transfer. We assume that the reactivity indicators in Eq. 3 do not depend on the number of electrons; this is reliable if the expression in Eq. 3 has already been iterated to convergence or, failing that, that the result of the first iteration (where all the reactivity indicators are computed for the isolated reagents) suffices. Typically, we would take $\Delta N_{A}+\Delta N_{B}=0$ , and then solve for the amount of charge transfer that minimizes the interaction energy. However, this leads to expressions that are far too complicated to analyze. Minimizing Eq. 3 requires solving a cubic equation, and even when there is a clear indication of which root should be taken, the resulting expressions are of little help. Hence, we need to introduce a second approximation, assuming that the dependence of Δ*N*_*A*_ and Δ*N*_*B*_ on the number of electrons is linear,

$$\begin{array}{l} \Delta N_{A}=\Delta N_{A}^{0}+d_{A}\delta \\ \Delta N_{B}=\Delta N_{B}^{0}+d_{B}\delta \end{array}$$ (17)

Here, $\Delta N_{A}^{0},\Delta N_{B}^{0}$ are just some convenient reference values used as starting points to expand the correct changes in particle numbers in *A* and *B*, respectively.

To calculate the chemical potential and hardness of the product, it is convenient to consider an excess charge on the products, namely (Miranda-Quintana R. A., 2017):

$$\Delta N_{A}+\Delta N_{B}=\delta$$ (18)

So this implies that

$$\begin{array}{l} \Delta N_{A}=\Delta N_{A}^{0}+d_{A}\delta \\ \Delta N_{B}=-\Delta N_{B}^{0}+\left(1-d_{A}\right)\delta \end{array}$$ (19)

Now we can substitute Eq. 19 in Eq. 14, consider an infinitesimal δ , and truncate at second order:

$$\begin{array}{c} \Delta U_{AB}\left[\Delta N_{A},\Delta N_{B}\right]=\delta \left\{\begin{array}{l} \frac{1}{2}c_{AB}\left(2d_{A}-1\right){\left(\Delta N_{A}^{0}\right)}^{3}+\Delta N_{A}^{0}\left[d_{A}\left(\eta_{A}+\eta_{B}\right)-2h_{AB}d_{A}+h_{AB}-\eta_{B}\right]+\\ \mu_{A}d_{A}-\mu_{B}d_{A}+\mu_{B}+{\left(\Delta N_{A}^{0}\right)}^{2}\left[-3\theta_{A}d_{A}+\theta_{A}+\theta_{B}\left(3d_{A}-2\right)\right] \end{array}\right\}+\\ \frac{1}{4}\delta^{2}\left\{\begin{array}{l} c_{AB}\left[6\left(d_{A}-1\right)d_{A}+1\right]{\left(\Delta N_{A}^{0}\right)}^{2}+2\eta_{A}{\left(d_{A}\right)}^{2}-4h_{AB}d_{A}\left(d_{A}-1\right)+\\ 2\eta_{B}{\left(d_{A}-1\right)}^{2}+4\Delta N_{A}^{0}\left[3{\left(d_{A}\right)}^{2}\left(\theta_{B}-\theta_{A}\right)+2\theta_{A}d_{A}-4\theta_{B}d_{A}+\theta_{B}\right] \end{array}\right\} \end{array}$$ (20)

Notice that we have omitted the terms that do not depend on δ . The linear and quadratic coefficients are the equations for the chemical potential and (twice the) hardness of the reaction product, respectively. Namely,

$$\mu_{AB}=\left\{\begin{array}{l} \frac{1}{2}c_{AB}\left(2d_{A}-1\right){\left(\Delta N_{A}^{0}\right)}^{3}+\Delta N_{A}^{0}\left[d_{A}\left(\eta_{A}+\eta_{B}\right)-2h_{AB}d_{A}+h_{AB}-\eta_{B}\right]+\\ \mu_{A}d_{A}-\mu_{B}d_{A}+\mu_{B}+{\left(\Delta N_{A}^{0}\right)}^{2}\left[-3\theta_{A}d_{A}+\theta_{A}+\theta_{B}\left(3d_{A}-2\right)\right] \end{array}\right\}$$ (21)

$$\eta_{AB}=\frac{1}{2}\left\{\begin{array}{l} c_{AB}\left[6\left(d_{A}-1\right)d_{A}+1\right]{\left(\Delta N_{A}^{0}\right)}^{2}+2\eta_{A}{\left(d_{A}\right)}^{2}-4h_{AB}d_{A}\left(d_{A}-1\right)+\\ 2\eta_{B}{\left(d_{A}-1\right)}^{2}+4\Delta N_{A}^{0}\left[3{\left(d_{A}\right)}^{2}\left(\theta_{B}-\theta_{A}\right)+2\theta_{A}d_{A}-4\theta_{B}d_{A}+\theta_{B}\right] \end{array}\right\}$$ (22)

These are the fundamental expressions of this manuscript, as they serve as the basis for our forthcoming analyses.

### 1.2 The maximum hardness principle

A key point in Eq. 17 is how to estimate $\Delta N_{A}^{0}$ and $d_{A}$ . Perhaps the simplest route is to just use the standard parabolic model result, thus:

$$\begin{array}{c} \Delta N_{A}^{0}=\frac{\mu_{B}^{0}-\mu_{A}^{0}}{\eta_{A}^{0}+\eta_{B}^{0}}\\ d_{A}=\frac{\eta_{B}^{0}}{\eta_{A}^{0}+\eta_{B}^{0}} \end{array}$$ (23)

In the case of the hardness, this leads to a relatively simple expression:

$$\eta_{AB}\approx {\left(\frac{1}{\eta_{A}^{0}+\eta_{B}^{0}}\right)}^{2}\left(\begin{array}{c} -\int \left({\left(\eta_{B}^{0}\right)}^{2}f_{A}^{\left(2\right)}\left(r\right)\Phi_{B}^{0}\left(r\right)+{\left(\eta_{A}^{0}\right)}^{2}f_{B}^{\left(2\right)}\left(r\right)\Phi_{A}^{0}\left(r\right)\right)dr\\ +\iint \frac{\left(\begin{array}{l} {\left(\eta_{B}^{0}\right)}^{2}\Delta \rho_{B}^{\left(\text{pol}\right)}\left[\Phi_{A}^{0},r\right]f_{A}^{\left(2\right)}\left(r^{\prime }\right)\\ +{\left(\eta_{A}^{0}\right)}^{2}\Delta \rho_{A}^{\left(\text{pol}\right)}\left[\Phi_{B}^{0},r\right]f_{B}^{\left(2\right)}\left(r^{\prime }\right) \end{array}\right)}{\left|r-r^{\prime }\right|}drdr^{\prime }\\ +2\eta_{A}\eta_{B}\iint \frac{f_{A}\left(r\right)f_{B}\left(r^{\prime }\right)}{\left|r-r^{\prime }\right|}drdr^{\prime }\\ +\frac{2\left(\mu_{A}^{0}-\mu_{B}^{0}\right)}{\eta_{A}^{0}+\eta_{B}^{0}}\iint \frac{\left(\begin{array}{l} \eta_{A}^{0}\left(2\eta_{B}^{0}-\eta_{A}^{0}\right)f_{A}\left(r\right)f_{B}^{\left(2\right)}\left(r^{\prime }\right)\\ -\eta_{B}^{0}\left(2\eta_{A}^{0}-\eta_{B}^{0}\right)f_{B}\left(r\right)f_{A}^{\left(2\right)}\left(r^{\prime }\right) \end{array}\right)}{\left|r-r^{\prime }\right|}drdr^{\prime }\\ +\left(\frac{{\left(\mu_{A}^{0}-\mu_{B}^{0}\right)}^{2}\left({\left(\eta_{A}^{0}\right)}^{2}-4\eta_{A}^{0}\eta_{B}^{0}+{\left(\eta_{B}^{0}\right)}^{2}\right)}{{\left(\eta_{A}^{0}+\eta_{B}^{0}\right)}^{2}}\right)\\ \times \left(\iint \frac{f_{A}^{\left(2\right)}\left(r\right)f_{B}^{\left(2\right)}\left(r^{\prime }\right)}{\left|r-r^{\prime }\right|}drdr^{\prime }\right)\\ +\eta_{A}^{0}\eta_{B}^{0}\left(\eta_{A}^{0}+\eta_{B}^{0}\right) \end{array}\right)$$ (24)

where, for the sake of completeness, we have reverted back to the original notation, showing all the contributions to the interaction hardness in terms of both reagents.

Since $\eta_{A}\eta_{B}>0$ , and the reaction energy tends to decrease when $h_{AB}$ increases (Eq. 15), it is straightforward to corroborate that the “Fukui function” pairing that minimizes the energy (cf. Eq. 15) also guaranties a maximum hardness value.

Analyzing the term corresponding to the electrostatic interaction of the dual descriptors, namely ${\left(\eta_{A}^{0}\right)}^{2}-4\eta_{A}^{0}\eta_{B}^{0}+{\left(\eta_{B}^{0}\right)}^{2}$ , is a bit more involved. Since the energy tends to decrease when $c_{AB}$ decreases (Eq. 16), the interaction between the dual descriptors that minimizes the energy will maximize the hardness if

$${\left(\eta_{A}^{0}\right)}^{2}-4\eta_{A}^{0}\eta_{B}^{0}+{\left(\eta_{B}^{0}\right)}^{2}<0$$ (25)

However, this will be true only when:

$$2-\sqrt{3}<\frac{\eta_{B}^{0}}{\eta_{A}^{0}}<2+\sqrt{3}$$ (26)

This might seem like an odd result, since at it (falsely) seems like it introduces an asymmetry between the reactants. However, because $\left(2-\sqrt{3}\right)\left(2+\sqrt{3}\right)=1$ , one can rearrange this equation so that the symmetry of the expression with respect to permutation of the reactant labels is clear: $2-\sqrt{3}<\frac{\eta_{B}^{0}}{\eta_{A}^{0}}<2+\sqrt{3}\Leftrightarrow 2-\sqrt{3}<\frac{\eta_{A}^{0}}{\eta_{B}^{0}}<2+\sqrt{3}$ . Notice that Eq. 26 means that the MHP will hold when the hardnesses of the reactants are not very different. This implies that there could be cases where minimizing the energy actually implies that the hardness will tend to decrease. Equation 26 is consistent with other results from the literature which indicates that in double-exchange reactions of acids and bases, the HSAB and DMB rules are be fulfilled only when the differences in hardness of the reactants are not too large (Cardenas and Ayers, 2013; Miranda-Quintana et al., 2018). This is not especially concerning as the restriction on the hardness values is rarely implicated. For example, excluding the (very hard) noble gas atoms, Eq. 26 is violated by very few atom pairs within the periodic table (Cárdenas et al., 2016), and the pairs that do violate the constraint (e.g. Cesium and Fluorine) are so extreme that there is little need for additional tools to elucidate their reactivity.

These results provide some support for the MHP, but they rely on the parabolic model (Eq. 23). We can obtain a more realistic estimate of $\Delta N_{A}^{0}$ and $d_{A}$ if we work instead with a simplified version of Eq. 14 where we neglect all cross-terms (i.e., $\Delta N_{A}\Delta N_{B},{\left(\Delta N_{A}\right)}^{2}\Delta N_{B},\Delta N_{A}{\left(\Delta N_{B}\right)}^{2},{\left(\Delta N_{A}\Delta N_{B}\right)}^{2}$ ). Thus, we are still working with a parabolic model, but now the descriptors have some information regarding the perturbation induced by the other reagent. Therefore now we will have

$$\begin{array}{c} \Delta N_{A}^{0}=\frac{\mu_{B}-\mu_{A}}{\eta_{A}+\eta_{B}}\\ d_{A}=\frac{\eta_{B}}{\eta_{A}+\eta_{B}} \end{array}$$ (27)

Now the expression for the energy reads:

$$\begin{array}{c} \Delta U_{AB}\left[\Delta N_{A},\Delta N_{B}\right]\approx \delta \left\{\begin{array}{l} -\frac{c_{AB}\left(\eta_{B}-\eta_{A}\right){\left(\mu_{A}-\mu_{B}\right)}^{3}}{2{\left(\eta_{A}+\eta_{B}\right)}^{4}}+\frac{h_{AB}\left(\eta_{B}-\eta_{A}\right)\left(\mu_{A}-\mu_{B}\right)}{{\left(\eta_{A}+\eta_{B}\right)}^{2}}+\\ \frac{\mu_{A}\eta_{B}+\mu_{B}\eta_{A}}{\left(\eta_{A}+\eta_{B}\right)}+\frac{{\left(\mu_{A}-\mu_{B}\right)}^{2}\left[\eta_{A}\left(\theta_{A}-2\theta_{B}\right)+\eta_{B}\left(\theta_{B}-2\theta_{A}\right)\right]}{{\left(\eta_{A}+\eta_{B}\right)}^{3}} \end{array}\right\}+\\ \frac{\delta^{2}}{4{\left(\eta_{A}+\eta_{B}\right)}^{4}}\left\{\begin{array}{l} c_{AB}\left(\eta_{A}^{2}-4\eta_{A}\eta_{B}+\eta_{B}^{2}\right){\left(\mu_{A}-\mu_{B}\right)}^{2}+\\ 2\eta_{A}\eta_{B}{\left(\eta_{A}+\eta_{B}\right)}^{3}+4h_{AB}\eta_{A}\eta_{B}\left(\eta_{A}+\eta_{B}\right)+\\ 4\left(\eta_{A}+\eta_{B}\right)\left(\mu_{A}-\mu_{B}\right)\left[\eta_{B}\theta_{A}\left(\eta_{B}-2\eta_{A}\right)-\eta_{A}\theta_{B}\left(\eta_{A}-2\eta_{B}\right)\right] \end{array}\right\} \end{array}$$ (28)

Hence

$$\eta_{AB}=\frac{1}{2{\left(\eta_{A}+\eta_{B}\right)}^{4}}\left\{\begin{array}{l} c_{AB}\left(\eta_{A}^{2}-4\eta_{A}\eta_{B}+\eta_{B}^{2}\right){\left(\mu_{A}-\mu_{B}\right)}^{2}+2\eta_{A}\eta_{B}{\left(\eta_{A}+\eta_{B}\right)}^{3}+4h_{AB}\eta_{A}\eta_{B}\left(\eta_{A}+\eta_{B}\right)\\ +4\left(\eta_{A}+\eta_{B}\right)\left(\mu_{A}-\mu_{B}\right)\left[\eta_{B}\theta_{A}\left(\eta_{B}-2\eta_{A}\right)-\eta_{A}\theta_{B}\left(\eta_{A}-2\eta_{B}\right)\right] \end{array}\right\}$$ (29)

In this case we can obtain support for the MHP in the same way as we did before: the coefficient of the $h_{AB}$ term is positive, therefore the “Fukui function pairing” that minimizes the energy also guaranties a maximum hardness value.

As for the dual descriptor interactions, now the MHP will be true if:

$$2-\sqrt{3}<\frac{\eta_{B}}{\eta_{A}}<2+\sqrt{3}$$ (30)

As we saw previously, this result also indicates that just maximizing the hardness might not always lead to more favorable interactions between various reagents. Only when the harder reactant is no more than ∼3.7 times higher than the hardness of the softer reagent might the formation of the hardest product will be favored.

### 1.3 The “|dμ| big is good” principle

Given the generally more complicated nature of the expressions involved in the treatment of the DMB principle, we will only consider the reference $\Delta N_{A}^{0}$ and $\nu_{A}$ presented in Eq. 27. Substituting these expressions into Eq. 20:

$$\begin{array}{c} \Delta \mu_{AB}=\frac{\left(\eta_{B}-\eta_{A}\right)\left(\mu_{A}-\mu_{B}\right)}{2\left(\eta_{A}+\eta_{B}\right)}\left\{1-\frac{c_{AB}{\left(\mu_{A}-\mu_{B}\right)}^{2}}{{\left(\eta_{A}+\eta_{B}\right)}^{3}}+\frac{2h_{AB}}{\left(\eta_{A}+\eta_{B}\right)}\right\}+\\ \frac{{\left(\mu_{A}-\mu_{B}\right)}^{2}\left[\eta_{A}\left(\theta_{A}-2\theta_{B}\right)+\eta_{B}\left(\theta_{B}-2\theta_{A}\right)\right]}{{\left(\eta_{A}+\eta_{B}\right)}^{3}} \end{array}$$ (31)

As it was the case in the last part of the discussion on the MHP, we will discard the cross-terms corresponding to the ${\left(\Delta N_{A}\right)}^{2}\Delta N_{B},\Delta N_{A}{\left(\Delta N_{B}\right)}^{2}$ factors, which means that we can write:

$$\Delta \mu_{AB}=\frac{\left(\eta_{B}-\eta_{A}\right)\left(\mu_{A}-\mu_{B}\right)}{2\left(\eta_{A}+\eta_{B}\right)}\left\{1-\frac{c_{AB}{\left(\mu_{A}-\mu_{B}\right)}^{2}}{{\left(\eta_{A}+\eta_{B}\right)}^{3}}+\frac{2h_{AB}}{\left(\eta_{A}+\eta_{B}\right)}\right\}$$ (32)

With this approximation, the energy model reduces to:

$$\begin{array}{c} \Delta U_{AB}\left[\delta \right]\approx -\frac{{\left(\mu_{A}-\mu_{B}\right)}^{2}}{2\left(\eta_{A}+\eta_{B}\right)}\left\{\frac{2{\left(\eta_{A}+\eta_{B}\right)}^{2}\left(\eta_{A}+\eta_{B}+2h_{AB}\right)-c_{AB}{\left(\mu_{A}-\mu_{B}\right)}^{2}}{2{\left(\eta_{A}+\eta_{B}\right)}^{3}}\right\}\\ \delta \left\{\begin{array}{l} -\frac{c_{AB}\left(\eta_{B}-\eta_{A}\right){\left(\mu_{A}-\mu_{B}\right)}^{3}}{2{\left(\eta_{A}+\eta_{B}\right)}^{4}}+\\ \frac{h_{AB}\left(\eta_{B}-\eta_{A}\right)\left(\mu_{A}-\mu_{B}\right)}{{\left(\eta_{A}+\eta_{B}\right)}^{2}}+\frac{\mu_{A}\eta_{B}+\mu_{B}\eta_{A}}{\left(\eta_{A}+\eta_{B}\right)} \end{array}\right\}+\\ \frac{\delta^{2}}{4{\left(\eta_{A}+\eta_{B}\right)}^{4}}\left\{\begin{array}{l} c_{AB}\left(\eta_{A}^{2}-4\eta_{A}\eta_{B}+\eta_{B}^{2}\right){\left(\mu_{A}-\mu_{B}\right)}^{2}+\\ 2\eta_{A}\eta_{B}{\left(\eta_{A}+\eta_{B}\right)}^{3}+4h_{AB}\eta_{A}\eta_{B}\left(\eta_{A}+\eta_{B}\right) \end{array}\right\} \end{array}$$ (33)

Here we have elected to explicitly include the constant (the δ -independent term):

$$\Delta U_{AB}=\Delta U_{AB}\left[0\right]\approx -\frac{{\left(\mu_{A}-\mu_{B}\right)}^{2}}{2\left(\eta_{A}+\eta_{B}\right)}\left\{\frac{2{\left(\eta_{A}+\eta_{B}\right)}^{2}\left(\eta_{A}+\eta_{B}+2h_{AB}\right)-c_{AB}{\left(\mu_{A}-\mu_{B}\right)}^{2}}{2{\left(\eta_{A}+\eta_{B}\right)}^{3}}\right\}.$$ (34)

Equations 32 and 34 can be rewritten as:

$$\Delta \mu_{AB}=\Delta \mu_{AB}^{\left(PP\right)}\left\{1-\frac{c_{AB}{\left(\mu_{A}-\mu_{B}\right)}^{2}}{{\left(\eta_{A}+\eta_{B}\right)}^{3}}+\frac{2h_{AB}}{\left(\eta_{A}+\eta_{B}\right)}\right\}$$ (35)

$$\Delta U_{AB}=\Delta U_{AB}^{\left(PP\right)}\left\{\frac{2{\left(\eta_{A}+\eta_{B}\right)}^{2}\left(\eta_{A}+\eta_{B}+2h_{AB}\right)-c_{AB}{\left(\mu_{A}-\mu_{B}\right)}^{2}}{2{\left(\eta_{A}+\eta_{B}\right)}^{3}}\right\},$$ (36)

where the (PP) index indicates that these are the expressions obtained using the Parr-Pearson parabolic model. (Parr and Pearson, 1983)

Without losing any generality, we can assume that *A* is the acid, namely: $\mu_{A}<\mu_{B}$ . Then, since, the terms in brackets in Eqs 35, 36 are always positive (cf. Eq. 16, proving the DMB is equivalent to showing that:

$$\left(\frac{\partial \Delta U}{\partial \Delta \mu }\right)>0\;\mathrm{when}\;\eta_{A}<\eta_{B}$$ (37)

$$\left(\frac{\partial \Delta U}{\partial \Delta \mu }\right)<0\;\mathrm{when}\;\eta_{A}>\eta_{B}$$ (38)

which can be rephrased as:

$$\mathrm{sgn}\left(\frac{\partial \Delta U}{\partial \Delta \mu }\right)=\mathrm{sgn}\left(\eta_{B}-\eta_{A}\right)$$ (39)

Closely following the strategy employed in previous approaches to DMB (Miranda-Quintana et al., 2018), we can take (notice that equivalent expressions for the change in reactant B can be obtained by simply exchanging the indices in the following equations)

$$\frac{\partial \Delta U}{\partial \Delta \mu }=\frac{\partial \Delta U}{\partial \mu_{A}}\frac{\partial \mu_{A}}{\partial \Delta \mu }+\frac{\partial \Delta U}{\partial \eta_{A}}\frac{\partial \eta_{A}}{\partial \Delta \mu }$$ (40)

resulting in:

$$\frac{\partial \Delta U}{\partial \Delta \mu }=T+T_{A},$$ (41)

where:

$$T=\left(\mu_{A}-\mu_{B}\right)\left\{\frac{2\left[{\left(\eta_{A}+\eta_{B}\right)}^{2}\left(\eta_{A}+\eta_{B}+2h_{AB}\right)-c_{AB}{\left(\mu_{A}-\mu_{B}\right)}^{2}\right]}{\left(\eta_{A}-\eta_{B}\right)\left[{\left(\eta_{A}+\eta_{B}\right)}^{2}\left(\eta_{A}+\eta_{B}+2h_{AB}\right)-3c_{AB}{\left(\mu_{A}-\mu_{B}\right)}^{2}\right]}\right\}$$ (42)

$$T_{A}=\left(\mu_{A}-\mu_{B}\right)\frac{\left[{\left(\eta_{A}+\eta_{B}\right)}^{2}\left(\eta_{A}+\eta_{B}+2h_{AB}\right)-2c_{AB}{\left(\mu_{A}-\mu_{B}\right)}^{2}\right]}{\left\{\begin{array}{l} -\eta_{A}\left[3c_{AB}{\left(\mu_{A}-\mu_{B}\right)}^{2}+2\eta_{B}^{2}\left(5h_{AB}+3\eta_{B}\right)\right]+\\ \eta_{B}\left[5c_{AB}{\left(\mu_{A}-\mu_{B}\right)}^{2}-2\eta_{B}^{2}\left(3h_{AB}+\eta_{B}\right)\right]+\\ 2\eta_{A}^{2}\left[\eta_{A}\left(h_{AB}-\eta_{B}\right)-\eta_{B}\left(h_{AB}+3\eta_{B}\right)\right] \end{array}\right\}}$$ (43)

It is easy to check that the sign of T only depends on the sign of $\eta_{B}-\eta_{A}$ , so we only need to show that $\mathrm{sgn}\left(T_{A}\right)=\mathrm{sgn}\left(\eta_{B}-\eta_{A}\right)$ . For this we only need to analyze the sign of the expression:

$$\begin{array}{c} {\tilde{T}}_{A}=\eta_{A}\left[3c_{AB}{\left(\mu_{A}-\mu_{B}\right)}^{2}+2\eta_{B}^{2}\left(5h_{AB}+3\eta_{B}\right)\right]-\\ \eta_{B}\left[5c_{AB}{\left(\mu_{A}-\mu_{B}\right)}^{2}-2\eta_{B}^{2}\left(3h_{AB}+\eta_{B}\right)\right]-\\ 2\eta_{A}^{2}\left[\eta_{A}\left(h_{AB}-\eta_{B}\right)-\eta_{B}\left(h_{AB}+3\eta_{B}\right)\right] \end{array}$$ (44)

which we can rewrite in a form that makes its dependence on the sign of $\eta_{B}-\eta_{A}$ more apparent,

$$\begin{array}{c} {\tilde{T}}_{A}=2\left(\eta_{B}-\eta_{A}\right)\left[-\eta_{A}^{2}\left(h_{AB}-\eta_{B}\right)+\eta_{B}^{2}\left(5h_{AB}+7\eta_{B}\right)+4\eta_{A}\eta_{B}^{2}-\frac{3}{2}c_{AB}{\left(\mu_{A}-\mu_{B}\right)}^{2}\right]-\\ \left[2c_{AB}\eta_{B}{\left(\mu_{A}-\mu_{B}\right)}^{2}+16\eta_{B}^{3}\left(\eta_{B}+h_{AB}\right)\right] \end{array}$$ (45)

Ignoring, for the moment, the term on the second line, DMB follows if:

$$-\eta_{A}^{2}\left(h_{AB}-\eta_{B}\right)+\eta_{B}^{2}\left(5h_{AB}+7\eta_{B}\right)+4\eta_{A}\eta_{B}^{2}-3c_{AB}{\left(\mu_{A}-\mu_{B}\right)}^{2}>0$$ (46)

This inequality is very likely to hold in most cases (particularly, in the weakly-interacting regime). The last three terms are always positive. The first term is likewise positive if that $h_{AB}<\eta_{B}$ _._ This can always be ensured by taking the initial separation of reagents to be sufficiently large (Yañez et al., 2021). For instance, for Fukui functions localized on two atomic sites separated by 5 Å, $h_{AB}$ is less than 3 eV. Hence, it is safe to assume that inequality 46) holds.

It remains to analyze the term $-\left[2c_{AB}\eta_{B}{\left(\mu_{A}-\mu_{B}\right)}^{2}+16\eta_{B}^{3}\left(\eta_{B}+h_{AB}\right)\right]$ , which cannot be factored in terms of $\eta_{B}-\eta_{A}$ .

These results largely point out to the validity of the DMB principle. The only potential incongruity comes in the form of the hard-to-factor terms in the expressions of ${\tilde{T}}_{A}$ and ${\tilde{T}}_{B}$ . However, these terms appear because we decided to perform a more rigorous mathematical treatment of the foundations of this principle. Should we have chosen to go with more qualitative arguments (as it was the case for the MHP, and some other discussions of reactivity principles), the evidence in favor of DMB would have been even stronger. Just note that, in accordance with Eqs 15, 16, the $h_{AB}>0$ and $c_{AB}<0$ conditions that guarantee a minimum interaction energy, are also the ones that, following Eq. 31, will tend to maximize |Δμ| . However, due to the central role of DMB in chemical reactivity, it is illustrative to perform a more detailed analysis of the conditions supporting its validity. Our analysis here indicates that the DMB principle is valid where certain terms become negligible. The confounding terms become negligible when reagents are sufficiently far apart, suggesting that failures of the DMB principle to predict reactivity are most likely to occur in cases where the activated complex in a chemical reaction is tightly bound or does not exist (e.g., barrierless reactions). This is consistent with the (already well-established) reduction of the efficacy of conceptual density functional theory in such cases, due mainly to the importance of higher-order terms in the perturbative expansion.

## 2 Summary

In this work we have shown that starting from an expression for the interaction energy between reactants deduced in the first part of this series of papers, it is possible to find a theoretical support for the maximum hardness principle. The main difference between this work and other related papers is that here the perturbation of one reactant on another is explicitly accounted for. In summary, the MHP is fulfilled if 1) the electrostatic interaction of the Fukui functions of the reactants is positive, (Berkowitz, 1987; Ayers P. W. and Levy M., 2000; Osorio et al., 2011), 2) the electrostatic interaction of the dual descriptors of the reactants is negative and, (Ayers et al., 2007; Cardenas et al., 2009b), 3) the relation between the hardnesses of the reactants is bounded by the inequality (30).

Similarly, we provided more arguments favoring the DMB principle, which unsurprisingly also seems to hold when we take in to account the full two-reagent picture. Contrary to the MHP case, establishing the validity of the DMB principle requires more caveats, and additional mathematical scrutiny is warranted. However, even simple qualitative discussion of the form of the expression for the change in reagents’ chemical potential support its validity, and also support the favorability of large Coulomb interactions between fragments’ Fukui functions and small Coulomb interactions between fragments’ dual descriptors.

Overall, the two-reagent model discussed in this and the previous contribution provides a more complete picture of chemical reactivity, encompassing several previous approaches, while also strengthening the arguments supporting several reactivity principles. Further applications of this framework are underway and will be presented elsewhere.

## Data Availability

The original contributions presented in the study are included in the article/Supplementary Material, further inquiries can be directed to the corresponding authors.
